# An American Elephant

**Published:** 1880-12

**Authors:** 


### AN AMERICAN ELEPHANT.

Recently William Hammond was engaged in digging a ditch on the farm of John H. Caylor, four miles southeast of Noblesville, on the Greenfield Pike, when he struck a part of the remains of what our local naturalists pronounce to have been an Elephas Americanus, or American elephant. The pieces found were two teeth and some bones. The teeth are each eight and one-half inches long from the front to the rear of the tooth, five and three-fourths inches broad, weighing each five and one-half pounds, and are in a good state of preservation. On the grinder the surface is flat and even, with raised edges running across from one side to the other. The bones are at the Citizens' Bank. The discoverer says he can find the complete set of bones of the skeleton.

